# Exploring factors relevant in the assessment of the return-to-work process of employees on long-term sickness absence due to a depressive disorder: a focus group study

**DOI:** 10.1186/1471-2458-12-103

**Published:** 2012-02-06

**Authors:** Anna Muijzer, Sandra Brouwer, Jan H Geertzen, Johan W Groothoff

**Affiliations:** 1 Department of Health Sciences, Community and Occupational Medicine, University Medical Center Groningen, University of Groningen, Groningen, The Netherlands; 2 Department of Rehabilitation Medicine, Center for Rehabilitation, University Medical Center Groningen, University of Groningen, Groningen, The Netherlands

## Abstract

**Background:**

Efforts undertaken during the Return-to-Work (RTW) process need to be sufficient in order to optimize the quality of the RTW process. The purpose of this study was to explore factors relevant to Return-to-Work Effort Sufficiency (RTW-ES) in cases of sick-listed employees with a Depressive Disorder (DD).

**Method:**

A case of a long-term sick-listed employee with a DD applying for disability benefits was used to gather arguments and grounds relevant to the assessment of RTW-ES. Two focus group meetings were held, consisting of Labor Experts working at the Dutch Social Insurance Institute. Factors were collected and categorized using the International Classification of Functioning, Disability and Health (ICF model).

**Results:**

Sixteen factors relevant to RTW-ES assessment in a case of DD were found, categorized in the ICF-model under activities (e.g. functional capacity), personal (e.g. competencies, attitude) and environmental domain (e.g. employer-employee relationship), or categorized under interventions, job accommodations and measures.

**Conclusions:**

This study shows that 16 factors are relevant in the assessment of RTW-ES in employees sick-listed due to DD. Further research is necessary to expand this knowledge to other health conditions, and to investigate the impact of these results on the quality of the RTW-ES assessment.

## Background

The assessment of the efforts made in the return-to-work (RTW) process is an important issue when considering the quality of the RTW process [1]. RTW efforts made in the RTW process include all activities undertaken by employee, employer or health professionals involved, i.e. medical specialists, occupational physicians and therapists [2].

In several countries the assessment of Return-to-Work Effort Sufficiency (RTW-ES) is performed as part of the evaluation of the RTW process in relation to the application for disability benefits [1]. The perspective of this assessment is that if the RTW process is designed effectively and the RTW efforts are sufficient, the chances of RTW are optimal, and in accordance with health status and work ability of the sick-listed employee.

In the Netherlands, the assessment of RTW-ES takes place prior to the assessment of functional and earning capacity as part of the disability evaluation, after two years of sickness absence [3]. The RTW-ES assessment is performed only when the employee has not fully returned to work after two years of sickness absence, but does have remaining work ability and is applying for disability benefits. A reintegration report forms the basis for the assessment. This report is written by the employer and employee, and includes a problem analysis, i.e. a mandatory description of the (dis)abilities of the employee by a physician of the Occupational Health Service (OHS) hired by the employer, an action plan, i.e. the plan designed to achieve work resumption, and the employee's opinion regarding the RTW process. Both employer and employee provide information about their opinion about the RTW process. The employer drafts the report, and the employee adds a form about (non-)acceptance of RTW process, procedures and outcome. This information is added to the reintegration report, on which the RTW-ES assessment is based. Also in the reintegration report are records of all interaction (i.e. interventions, conversations and agreements) between the parties involved in the RTW process (e.g. employer, employee, physicians, specialists). An example of such an interaction is a request for professional advice in the case of suspected insuffiency of efforts during the RTW process. This request can be made by both the employer and employee.

This quality of the RTW process is assessed by Labor Experts (LE's) working at the Social Insurance Institute (SII, National Institute of benefit Schemes, Uitvoeringsinstituut Werknemersverzekeringen (in Dutch)). LE's are specialists in the field of vocational rehabilitation, and after graduating, have followed a one to two year intensive postacademic in-company training. These LE's assess whether all opportunities for RTW have been investigated and seized (if applicable), and whether the conditions for RTW have been optimal. Moreover, the assessment focuses on which efforts can still be undertaken to improve chances of RTW. The LE's have the opportunity to consult a Social Insurance Physician (SIP), and can invite the employer and employee to provide more information by phone, letter or face-to-face contact.

Assessing an outcome such as the efforts made in the RTW process is an elaborate and complicated decision-making process, in which relevant factors are regarded implicitly [1,4]. In the Netherlands, a description about the assessment procedure of RTW efforts is available and described in a guideline for the LE's [3]. This guideline is an important instrument to ensure quality of the assessment and can be seen as a logical step in the process of professionalisation and quality assurance by LE's. However, this guideline consists mostly of information about the procedural aspects of the assessment, does not include an extensive list of factors which are relevant to the assessment, and is not based on scientific evidence. Up to date, studies on the sufficiency of RTW efforts are scarce [2], and little is known about the factors relevant to the assessment of RTW-ES.

A major cause of sickness absence and work disability is Depressive Disorder (DD) [5-7]. In the Netherlands, up to one-third of disability benefits in the Netherlands are awarded because of mental health conditions [8]. Of these mental health conditions, depressive disorder is the main cause of sickness absence (34%) [9], and has a more negative prognosis for RTW than other common mental health conditions [10].

To assess if the RTW process is designed effectively and the RTW efforts are sufficient in accordance with health status and work ability, it is of utmost importance to gather more explicit information about the factors relevant in the RTW-process of the sick-listed employee with depressive disorder applying for disability benefits. Factors were collected and categorized using the International Classification of Functioning, Disability and Health (ICF model). By analyzing our results within the ICF model we aim to use a comprehensive framework. Using this well-known categorization system also facilitates the connection to existing and future literature. This way, our approach could help to improve comparability.

Recently, we have investigated factors relevant to RTW-ES in a focus group study in workers sick-listed with Chronic Low Back Pain (CLBP) [4]. Now that we have investigated factors relevant to the assessment of RTW-ES in employees with CLBP, expanding this knowledge to include other health conditions is crucial. Knowing which factors are relevant in the assessment of RTW-ES by means of research and including this kind of information in the existing protocol will optimize not only the transparency and reliability but also the validity of the assessment. This optimized assessment should benefit the professionals assessing RTW-ES directly, and should benefit other stakeholders involved in the RTW process (e.g. employer, employee and health professionals) indirectly.

Therefore, the aim of this study is to explore factors relevant to RTW-ES in a case of a long-term sick-listed employee with Depressive Disorder applying for disability benefits, by means of focus group research.

## Methods

The study was designed as a focus group study with two separate groups which had two meetings each, using the same case vignette.

### Focus groups

The focus groups consisted of Labor Experts (LE's) working at the Social Insurance Institute (SII) in the Netherlands. We aimed at composing two focus groups of five to eight LE's. A minimum of five LE's in each group is necessary to ensure response diversity, and a maximum of eight LE's to facilitate discussion later in the focus group process. A total of 32 LE's were contacted by SII staff members, 16 from SII's in the northern region, 16 from SII's in the central region of the Netherlands. LE's were selected by the staff members for their expertise in the assessment of RTW-ES (one to six years experience), and to include members of all SII offices of the region. If the LE's agreed, the researcher (AM) contacted them and explained the study, and asked for their participation.

**Figure 1:**
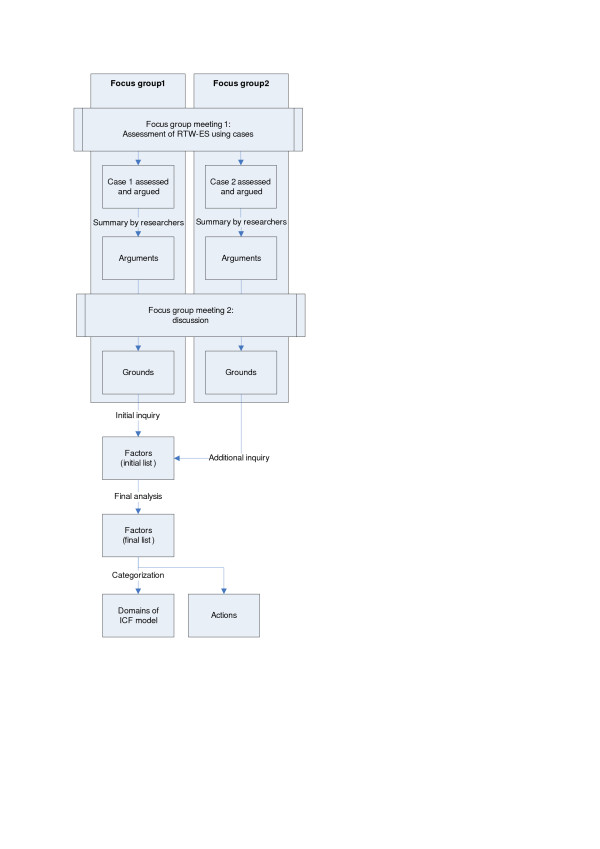
**Overview of procedure to make factors relevant to Return-to-work Effort Sufficiency explicit by means of focus groups**.

Each focus group had two meetings, where a case of RTW-ES was introduced. Both focus groups assessed the same case. The results from the second focus group were used to confirm and add to the findings of the first focus group. The procedure will be described in detail below (see also Figure 1).

### Case

The case was selected by two of the authors (AM and SB), with the help of five LE's. These LE's acted as an expert group and did not participate in the focus groups. The case was selected to reflect a realistic situation on the basis of a well-defined RTW process and outcome, resemblance to daily practice for the assessors, and sufficiency of the information in the reintegration report. The case represented an employee on sickness absence for two years due to a DD (ICD-10 code F32), which was diagnosed by a physician. The employee had not returned to work fully, showed no comorbidity (e.g. other diseases causing or prolonging the sickness absence), and had been available for RTW interventions (e.g. had not been institutionalized for a prolonged period of time).

The case was about a 47 year old male with secondary vocational education, working in horticulture. The employee had been working in a small company as middle management for six years. The work ability assessment by the occupational physician revealed that the employee was still suitable for his original job, but that RTW should be gradual and supported. The company did offer this temporary, adjusted work, and facilitated coaching. After one year, the employer had consulted experts from the SII, which advised that the coaching trajectory had been sufficient so far. In the second year, the employee suffered from several relapses, and a conflict between employer and employee emerged.

### Assessment

#### First focus group meeting = collecting the arguments

During the first meeting, a group of LE's was asked to assess individually the RTW efforts in the case presented. The procedure used matches the standard procedure at the Dutch SII, in which the LE receives the report made in the RTW process and the instruction to assess the RTW efforts. During the assessment, the LE's had access to their usual sources of information (e.g. legislation, guidelines, etc.). They were not allowed to consult each other or other LE colleagues. The LE's were given the opportunity to contact a fictitious employee, employer, physician of the OHS, and a physician of the SII. These roles were all performed by LE's, who had prepared their roles and had contacted physicians for advice and further information if they played the role of physician. The actors were provided with information which could be shared, and included a description of the behavior which was displayed. This standard procedure closely resembles the standard procedure used in the Dutch SII's when assessing RTW-ES.

In addition, the LE's received a clear instruction on the procedures of the day, and were asked to answer two questions. The questions were aimed at gathering information about 1) arguments used for deciding about the sufficiency of the RTW efforts, and 2) the decision outcome (sufficiency of RTW efforts). In order to analyze the data gathered per group, the authors (AM and SB) inventorized the arguments mentioned by the LE's, and also gathered information about who mentioned each argument. In order to collect the underlying grounds behind the arguments, a second meeting with the LE's took place.

#### Second meeting-collecting the underlying grounds

During the second meeting, the LE's were invited to participate in a discussion session. This meeting took about 4 h, and was chaired by a (senior) LE, with assistance of two of the authors (AM and SB). During the meeting, the participants were asked to explain why their arguments are relevant to the assessment of RTW efforts, thereby revealing the underlying ground of the argument. The ground is the underlying reason for mentioning the argument, and will make knowledge and experience more explicit [11]. The other participants were asked if they agreed with each ground provided, and were then asked to provide other grounds if available.

The researchers analyzed the grounds produced in the first focus group and collected factors from these grounds. All the words mentioned in the grounds have been considered, thereby collecting aspects relevant to the assessment of RTW-ES. Next, the grounds from the second focus group were analyzed to confirm factors found in the first focus group and identify additional factors. Finally, the authors (AM and SB) discussed all factors with each other in order to identify universal phrasing and correspondence between the factors. If AM and SB did not agree on the phrasing, other authors (JHBG and JWG) were consulted.

The factors were then categorized in accordance with the ICF model [12], into three domains: 1) activities, 2) personal, and 3) environmental.

### Ethics and consent

According to the Dutch Medical Research Involving Human Subjects Act (WMO), approval is not necessary for this focus group study. The professionals' opinions were collected with their consent, without any requirement to follow altered rules of behavior. No real patients were involved in the study, and the anonymized, altered case used in this study was made available by the SII.

## Results

The first focus group consisted of eight LE's, of which seven attended both meetings. The second focus group consisted of five LE's, of which all attended both meetings. One participant could not attend because of illness. Seven out of twelve LE's were male, and the LE's had between one-and-a-half and five years of experience in assessing RTW-ES.

### Arguments and underlying grounds

During the first meeting, the members of the first focus groups each assessed the case assigned to their focus group. The authors (AM and SB) summarized 49 arguments. An example of such an argument is 'The communication was not optimal'. During the second meeting, the LE's were asked to elaborate on the reason for mentioning this argument; the underlying grounds. In this example, the ground was 'Communication quantity is not necessarily an indicator of communication quality'. By exploring the arguments mentioned in relation to the case 48 underlying grounds were collected. The members of the second focus group provided 19 arguments, from which 23 underlying grounds were collected.

In order to collect factors relevant to RTW-ES, the authors (AM and SB) analyzed the grounds collected in the first focus group, and determined 16 factors and 3 subfactors (see Table 1). The second focus group did not yield any factors which were not mentioned by the first focus group. The resulting factors are described in relation to the ICF model in Table 1, including examples of grounds mentioned in relation to these factors.

**Table 1:** Grounds and factors relevant to Return-to-work Effort Sufficiency in depressive disorder according to focus groups, sorted using the International Classification of Functioning, Disability and Health (ICF) model

Factor	Ground example
**Activities**	

Functional capacity	"The type and severity of limitations are important to assess suitability for work"

*Job demands *vs. *functional capacity*	"The suitability of the employee's own work determines the RTW goal"

**Personal**	

Competencies	"The chances of RTW are higher in work which matches the competencies of the employee"

Attitude	"The attitude of the employee determines the progress of the RTW process"

Self-efficacy	"Fear of RTW can lead to inadequate RTW behaviour of the employee"

Illness perception	"A negative illness perception can delay the RTW process"

**Environmental**	

Work-relatedness of sickness absence	"The focus of the RTW process is determined by the work-relatedness of the absence"

Job availability	"The possibilities at the previous employer co-determine the chances of RTW"

*Employer size*	"Employer size indicates the possibilities for the employee to return to his/her original employer"

Employer attitude	"The employer has to support the employee in RTW at his/her company"

Relationship employer/employee	"The relationship between employer and employee can promote the RTW process"

*Communication quality and quantity*	"Communication quantity is not necessarily an indicator of communication quality"

**Interventions**	

Training/education	"Interventions such as job training can optimize RTW"

Job offerings	"An offered job with the prospect of returning to the original job increases the chance of acceptance by the employee"

Professional advice	"Professional advice can influence the RTW process"

**Job accommodations**	

Temporary/modified duty	"Offering temporary work can influence the chance of RTW, because the employee does not leave the work force"

Change of employer	"If RTW is prolonged and chances of RTW are reduced, return to a different employer should be investigated"

**Measures**	

Research/assessment	"The employer has to investigate the possibilities at his/her company"

Monitoring	"The progress of the employee should be monitored by the employer"

### Factors related to RTW-ES in depressive disorder

Factors related to RTW-ES in the activity domain of the ICF are the factor 'functional capacity', and its subfactor 'job demands vs. functional capacity'. Related to the personal domain are 'competencies', 'attitude', 'self-efficacy' and 'illness perception'.

Relevant environmental factors are 'work-relatedness of the sickness absence', 'job availability', with a subfactor 'employer size'. Other factors related to the environmental domain are 'employer attitude', 'relationship between employer and employee', and the subfactor 'communication quality and quantity'.

Seven factors describe actions rather than a situation, and could not be fitted within the ICF model. These factors are categorized under interventions, job accommodation and measures. Interventions are 'training/education', 'job offerings', and 'professional advice'. Job accommodations are 'temporary/modified duty', and 'change of employer'. Measures are 'research/assessment', and 'monitoring'.

## Discussion

Sixteen factors and three subfactors related to RTW-ES were identified after analyzing arguments and grounds of LE's derived from a case with an employee sick-listed due to depressive disorder. Of these 16 factors, 9 were fitted within the domains of the ICF model: 'functional capacity' and 'job demands vs. functional capacity' (activity domain), 'competencies', 'attitude', 'illness perception', and 'self-efficacy' (personal factors); 'work-relatedness of sickness absence', 'job availability', 'employer size', 'employer attitude', 'relationship between the employer and employee', 'communication quality and quantity' (environmental factors). Seven factors did not fit within the ICF model because they describe actions undertaken during the RTW process. These factors are categorized as interventions ('training/education', 'job offerings', 'professional advice'), job accommodations ('temporary/modified duty', 'change of employer'), and measures ('research/assessment', 'monitoring').

A comparison of the factors relevant in the assessment of RTW-ES in employees sick-listed due to a DD and due to CLBP [4] shows that 16 factors and 2 subfactors are relevant to both health conditions. The three factors which were only considered relevant in cases of CLBP are 'age', 'educational level' and 'tenure'. One sub-factor, 'suitability of own work', was considered relevant in the cases of CLBP only, and another sub-factor, 'reorganization', was considered to be relevant only in the case of DD.

When RTW processes in physical and mental health conditions are compared, initial RTW interventions mainly focus on reducing the symptoms of the health conditions [13]. In physical health conditions the focus is on reducing the symptoms in physical functioning, in mental health conditions the focus is on reduction of stress, coaching and supervisor support [14,15]. However, in long-term sickness absence, RTW trajectories in both health conditions become more similar because psychosocial factors rather than symptoms influence the RTW process. When long-term conditions are considered, disease specificity is significantly reduced [16-18], which is consistent with our comparison of DD and CLBP. Our findings of similarity between the factors relevant to the assessment of RTW-ES seem to be in line with the literature on factors relevant in the RTW process of employees on long-term sickness absence. However, these differences between factors relevant in cases of DD and CLBP could be attributed to case differences. For example, the difference in relevance of the factor 'age' might be contributed to differences in the cases used. In the cases of CLBP, the employees in question were older than in the case regarding DD (50 and 57 vs. 47 years old). The chance of RTW decreases when employees are over 45 years of age [19]. The participating LE's in our study explained in the CLBP cases that they expect the employer to undertake more efforts when the age of the employee is increased [4]. In the case of DD it could be that LE's do not consider an age of 47 a relevant factor for RTW-ES. According to literature, age is important to RTW in both mental and physical health conditions [13]. However, the relation between age and time to RTW is less pronounced in mental health conditions. In a study of Koopmans et al. (2008) it was found that the factor 'age' is less relevant in mental health conditions, because time to RTW is already high in younger age groups [17]. However, some factors could be considered disease-specific. It could be that for example reorganization is only relevant in DD because reorganizations could cause a more stressful environment, thereby decreasing the chances of RTW in DD, but this might not have an added effect in CLBP. Future research is necessary to investigate the influence of case differences and disease specificity in the assessment of RTW-ES.

The relevance of this study lies in its unique topic of interest. The assessment of RTW-ES is an important part of the RTW process, but little information and no evidence-based guidelines are available. Investigating factors relevant in the assessment of RTW-ES is crucial for the optimization of the quality of the RTW process. Knowing which factors are relevant in the assessment of RTW-ES by means of research and including this kind of information in the existing protocol will optimize not only the transparency and reliability but also the validity of the assessment. Based on these results, a multifactorial approach to the assessment of RTW-ES is essential. A guideline focusing on all relevant factors could improve the quality of the assessment of RTW-ES and could support professionals involved in the RTW process in designing and monitoring the RTW process and the activities undertaken during this process. This optimized assessment should therefore not only benefit the LE's assessing RTW-ES, but also other stakeholders involved in the RTW process, e.g. employer, employee and health professionals. Considering the importance of the assessment of RTW-ES, this optimization is essential.

The main strength of this study lies in its investigation of this unknown territory by means of focus group research. Focus groups have proven to be an effective method to gather information about implicit knowledge of professionals [11,13,20,21]. When literature is lacking, expert knowledge is considered an appropriate means of collecting evidence. Also, we have used two focus groups, to ensure a minimal amount and diversity of response. The two focus groups yielded a different number of arguments, however, both groups touched the same major issues when discussing the arguments and underlying grounds.

Moreover, during the assessments, additional information was presented by actors in order to reflect a more realistic situation. We agree that this method of presenting additional information could be a source of variation. However, we have chosen for this method because it reflects a more realistic situation, and we wanted the assessment to be as close to the actual situation as possible.

A possible limitation of this study is the use of only one case. Also, the case depicted a specific case of DD, while there are many appearances of DD. However, the effect of the use of this single case is unknown. While the use of more cases or different cases might have yielded different results, the majority of the results which have been found in relation to the cases about CLBP have been replicated in this study.

Future research should focus on the reproducibility of this study in a different context by investigating different cases, with different health conditions, DD appearances, or employee characteristics (e.g. gender). Using different focus groups and legislatory context could improve the generalizability of the results and provide more insight into the assessment of RTW-ES. Also of interest for further research would be the effect of the use of the factors on the reliability of the assessment. Providing a list of the relevant factors could increase the homogeneity and thereby the quality of the assessment of RTW-ES.

## Conclusions

In conclusion, this study shows that 16 factors are relevant in the assessment of RTW-ES in employees sick-listed due to a DD. This information is crucial considering the importance of the assessment of RTW-ES. Further research is required to explore factors relevant to RTW-ES in other health conditions, and to investigate the impact of these results on the quality of the assessment of RTW-ES.

## Competing interests

This study was funded by a SIG grant (Stichting Instituut Gak), the Netherlands. The authors declare no competing interests.

## Authors' contributions

AM carried out the study and has drafted the manuscript. SB helped carry out the study, participated in its design and coordination, and helped to draft the manuscript. JHBG participated in the design of the study and helped to draft the manuscript. JWG participated in the design of the study and helped to draft the manuscript. All authors read and approved the final manuscript.

## Pre-publication history

The pre-publication history for this paper can be accessed here:

http://www.biomedcentral.com/1471-2458/12/103/prepub
